# *Piwi* Genes Are Dispensable for Normal Hematopoiesis in Mice

**DOI:** 10.1371/journal.pone.0071950

**Published:** 2013-08-23

**Authors:** Mona J. Nolde, Ee-chun Cheng, Shangqin Guo, Haifan Lin

**Affiliations:** 1 Yale Stem Cell Center, Yale University School of Medicine, New Haven, Connecticut, United States of America; 2 Department of Cell Biology, Yale University School of Medicine, New Haven, Connecticut, United States of America; 3 Department of Genetics, Yale University School of Medicine, New Haven, Connecticut, United States of America; Hong Kong University of Science and Technology, China

## Abstract

Hematopoietic stem cells (HSC) must engage in a life-long balance between self-renewal and differentiation to sustain hematopoiesis. The highly conserved PIWI protein family regulates proliferative states of stem cells and their progeny in diverse organisms. A Human piwi gene (for clarity, the non-italicized “piwi” refers to the gene subfamily), *HIWI (PIWIL1)*, is expressed in CD34^+^ stem/progenitor cells and transient expression of *HIWI* in a human leukemia cell line drastically reduces cell proliferation, implying the potential function of these proteins in hematopoiesis. Here, we report that one of the three piwi genes in mice, *Miwi2 (Piwil4)*, is expressed in primitive hematopoetic cell types within the bone marrow. Mice with a global deletion of all three piwi genes, *Miwi*, *Mili*, and *Miwi2*, are able to maintain long-term hematopoiesis with no observable effect on the homeostatic HSC compartment in adult mice. The PIWI-deficient hematopoetic cells are capable of normal lineage reconstitution after competitive transplantation. We further show that the three piwi genes are dispensable during hematopoietic recovery after myeloablative stress by 5-FU. Collectively, our data suggest that the function of the piwi gene subfamily is not required for normal adult hematopoiesis.

## Introduction

Hematopoiesis is driven by a rare population of cells called hematopoietic stem cells (HSCs). These cells harbor the ability to repopulate all cell lineages within the blood system, throughout the lifetime of an animal. Human hematopoietic stem and progenitor cells are characterized by their expression of CD34 [1]–[4]. Therefore, CD34 expression can serve as an indicator of self-renewal capacity. Previously, it was shown that one of the four human homologs of *Drosophila piwi*, *PIWIL1 (a.k.a., HIWI*), is expressed in CD34^+^ cells of normal human peripheral blood [5]. This expression is quickly lost when these cells are differentiated to the myeloid lineage in culture [5], suggesting that piwi expression is correlated with undifferentiated cell states within the human blood system. In addition, when HIWI was transiently expressed in the leukemic cell line KG1, these cells were drastically reduced in their proliferation ability [5]. Yet, in mice, piwi gene expression was found to correlate with increased proliferative capacity in cells of the blood system, as ectopic expression of the *PIWIL2* homolog (a.k.a., *Mili*), one of the three piwi genes in mice, promoted expansion of hematopoetic cells in culture [6]. These interesting, but somewhat conflicting observations, point to the pressing need for a loss-of-function study to clearly define the role, if any, of PIWI proteins in hematopoiesis.

PIWI proteins are known to bind small non-coding RNAs, called PIWI-interacting RNAs (piRNAs) [7]–[16] and have highly conserved roles in germline development, gametogenesis, transposon silencing, and epigenetic regulation. Our studies of the piwi genes in *Drosophila*, *C. elegans*, mice, and humans, together with studies of piwi-like genes in *Arabidopsis*, by others, indicate that PIWI subfamily proteins represent the only known class of proteins with a highly conserved function for stem cell self-renewal in both animal and plant kingdoms [17]. Although the most well studied role for piwi genes is in the germline, mammalian piwi homologs are also expressed in a range of somatic tissues [18], [19].

Understanding molecular mechanisms underlying hematopoiesis has important clinical implications. The overexpression of mammalian PIWI is highly correlated to pre-cancerous hematopoietic stem cells [6], gastric cancers [20], and seminomas, a germline-derived testicular cancers [21]. Additionally, human piwi transcripts are expressed in a range of cancer cell lines and in cancer tissue samples [5], [20]–[29]. Furthermore, a recent study gave evidence that the P16INK4a tumor suppressor, a functional unit of the INK4/ARF locus, is directly regulated by the human *PIWIL4* gene. The promoter of P16INK4a locus contains multiple piRNA sites that, when deleted, cause mis-regulation of P16INK4 protein [19]. The INK4/ARF genomic region is needed during normal blood development to facilitate the cell death response of bone marrow progenitor cells following oncogenic insult and is commonly deleted in leukemia [30]. Together, these findings open the possibility that PIWI proteins might play important roles in multiple stem cell driven tissues, including the blood system. However, overexpression studies, either in normal or cancerous tissues, cannot define a role of a gene during normal development. Therefore, the requirement of PIWI proteins in hematopoiesis, remains to be established by loss-of-function studies.

To investigate a possible function of piwi genes in hematopoiesis, we created a triple knockout mouse model in which all three piwi genes, *Miwi* (*Piwil1*), *Mili* (*Piwil2*), and *Miwi2* (*Piwil4*), are deleted in the genome. Our analysis of the triple knockout mutants illustrates that piwi genes are not required for normal adult hematopoiesis.

## Materials and Methods

### Ethics Statement

Animal experiments in this study were carried out in accordance with the Animal Use Protocols as approved by the Institutional Animal Care and Use Committee, Yale University (IACUC Protocol number: 2009–11087). Mouse blood collection was performed under approved anesthesia, and all efforts were made to minimize suffering.

### Piwi knockout mice strains

Mice carrying deletions for single piwi genes were generated as previously described in refs. [31]–[33]. Triple knockout mice on a mixed B6129S genetic background were generated by crossing triple heterozygous males with triple knockout females. Genotypes were verified using multiplex PCR for each gene with the following primers, *Miwi/Piwil1*: 5′- TTGAAAAGCATTGAACACCATAAG –3′ and 5’- AGGTTG CTGGCTCTGCTCATGAAT– 3′and 5′– GACAGAAAATGACTGGCCGAGCCC –3′ (wild-type  = 400 bp; knockout  = 250 bp); *Mili/ Piwil2*: 5′– CGGCAGAGTGCAGTGAAGTTGG –3′ and 5′– AAAGGAATGATGCACTTGAGGG– 3′ and 5′– GCTCCAGACTGCCTTGGG –3′ (wild-type  = 239 bp; knockout  = 100 bp); *Miwi2/Piwil4*: 5′- AGTACCTTCCAAGTGGTG –3′ and 5′- GTCCACCATCACCAGAAG –3′ and 5′- CCTACCCGGTAGAATTGACC– 3′ and 5′- CAGCAACAATCATGCTAGA –3′ (wild-type  = 540 and 147 bp; knockout  = 300 bp).

### Bone marrow transplantation and 5FU treatment

For competitive repopulation studies, 1×10^6^ CD45.2 donor and 1×10^6^ CD45.1 competitor total nucleated bone marrow cells were mixed and injected into the tail veins of lethally irradiated CD45.1 B6 Ly5.2/Cr recipient mice treated with 9Gy dose via Cesium Irradiator. Hematopoietic recovery and lineage reconstitution were followed by serial analysis of peripheral blood beginning at 5 weeks post-transplantation. Peripheral blood was collected by retro-orbital or tail vein bleeding methods. Enucleated red blood cells were lysed with BD FACS Lysing Solution (BD Biosciences) following manufacturer's protocol and remaining cells were stained with antibodies to detect donor derived cells and committed lineages: CD45.2-FITC, B cells (B220-APC), T cells (CD3-PE Cy5), Myeloid (CD11b-PE). Flow cytometry was done on either a LSRII (BD) or a FACSCalibur (BD). Five week-old B6 Ly5.2/Cr (strain 01B96) recipient mice were purchased from the National Cancer Institute Mouse Repository (Frederick) and used within two-weeks for transplantation experiments. All animal studies were carried out as approved by the Yale University Institutional Animal Care and Use Committee.

For 5FU treatment, recipient mice were injected at 20 weeks post-competitive transplant (as described above) via intraperitoneal route with 25 mg/ml 5FU at a dose of 150 mg/kg. Recovery from HSC stress was monitored by serial sampling of peripheral blood subjected to Complete Blood Counts (CBC) and FACS analysis of committed lineages, as described above.

### Quantitative PCR

For Real-time quantitative PCR, total mRNA was extracted from FACS sorted bone marrow cells using either RNeasy Plus Mini kit (Qiagen) or RNAqueous-Micro Kit (Ambion). Mouse testis RNA was extraction with Trizol Reagent (Invitrogen Life Technologies) following manufacturer's protocol. cDNA was prepared using High-Capacity cDNA Reverse Transcription Kit (Applied Biosystems) and real-time quantitative PCR reactions were performed on a Biorad cycler using SybrGreen detection using the following primers for *Gapdh*: 5′- CCAGGAGCGAGACCCCACTAACA -3′ and 5′- TTCACACCCATCACAAACAT -3′ (177 bp); *Miwi2/Piwil4*: 5′- CTGACCCGGACCTTGAATAA -3′ and 5′- CGACCACCACATTCTTGTTG -3′ (175 bp).

### Cell sorting and flow cytometry

Bone marrow cells were obtained from hind limbs of mice and subjected to red blood cell lysis with BD Pharm Lyse (BD Biosciences), following manufacturer’s protocol. For cell sorting, lineage depletion for isolation of HSC and progenitor cell populations was performed by immuno-magnetic selection using Mouse Hematopoietic Progenitor (Stem) Cell Enrichment Set-DM (BD Biosciences) and a BD IMag Cell Separation Magnet. Following depletion, cells were stained simultaneously with Lineage Cell Detection Cocktail-Biotin (Miltenyi Biotec) and an antibody mix containing Sca-PE, cKit-APC, CD34-FITC, IL7Ra-PacBlue, CD16/32-PeCy7 (Progenitors) or Sca-PE, cKit-APC, CD34-FITC, CD48-PacBlue, CD150-PeCy7 (HSC), followed by a streptavidin-PerCP secondary antibody alone. Immunophenotypes were defined as described in reference [34]: HSC (Lin^−^cKit^+^Sca^+^CD48^−^CD150^+^), MEP (Lin^−^cKit^+^Sca^−^CD34^−^CD16/32^−^), CMP (Lin^−^cKit^+^Sca^−^CD34^+^CD16/32^−^), and GMP (Lin^−^cKit^+^Sca^−^CD34^+^CD16/32^+^). Bone marrow cells used for *Miwi2 (Piwil4)* real-time PCR were stained in a single tube, following lineage depletion, with an antibody cocktail including Lineage Cell Detection Cocktail-Biotin (Miltenyi Biotec), Sca-Alexa-647, cKit-APCH7, CD150-PECY5, CD105-PECY7, and CD16/32-FITC. Immunophenotype for these cells was defined as described in reference [35]: HSC (Lin^−^cKit^+^Sca^+^CD150^+^CD105^+^), multi-potent progenitors (MPP; Lin^−^cKit^+^Sca^+^CD150^−^CD105^−^), and GMP (Lin^−^cKit^+^Sca^−^CD150^−^CD16/32^+^). Lineage positive cell types were purified without lineage depletion using the following antibodies: B cells (B220-PE), T cells (CD3-PE), Macrophages (Gr1-PE, CD11b-FITC), Granulocytes (7/4-PE, Gr1-FITC). For Flow cytometry analysis, lineage depletion was not used for HSC and progenitor cell analysis. Cell sorting was done on a FACS Aria Cell Sorter (BD) and Flow cytometry was done on a LSRII (BD). All analysis of FACS data was done using FloJo Version 8.0 (TreeStar) software.

### Colony-Forming Unit Assay

A methylcellulose-based assay was performed whereby bone marrow was isolated from hind limbs of control or *Piwi* triple knockout mice. A total of 1×10^4^ whole bone marrow cells were mixed with Methocult GF M3434 (StemCell Technologies) containing SCF, IL-3, IL-6, and Erythropoietin. Cells from each mouse were plated in triplicate onto 35mm dishes and incubated at 37°C, 5% CO_2_ with high humidity. Colonies were scored after seven to ten days by counting total colony numbers for each replicate plate.

### Complete Blood Counts (CBC)

Total peripheral blood was collected by retro-orbital or tail vein bleeding into Capillary Blood Collection Tubes with EDTA (BD). CBC analysis was done using a standard mouse setting on a Hemavet 950FS (DREW Scientific, Inc.).

## Results

### A piwi gene, *Miwi2*, is expressed in the adult hematopoietic cells

**Figure 1:**
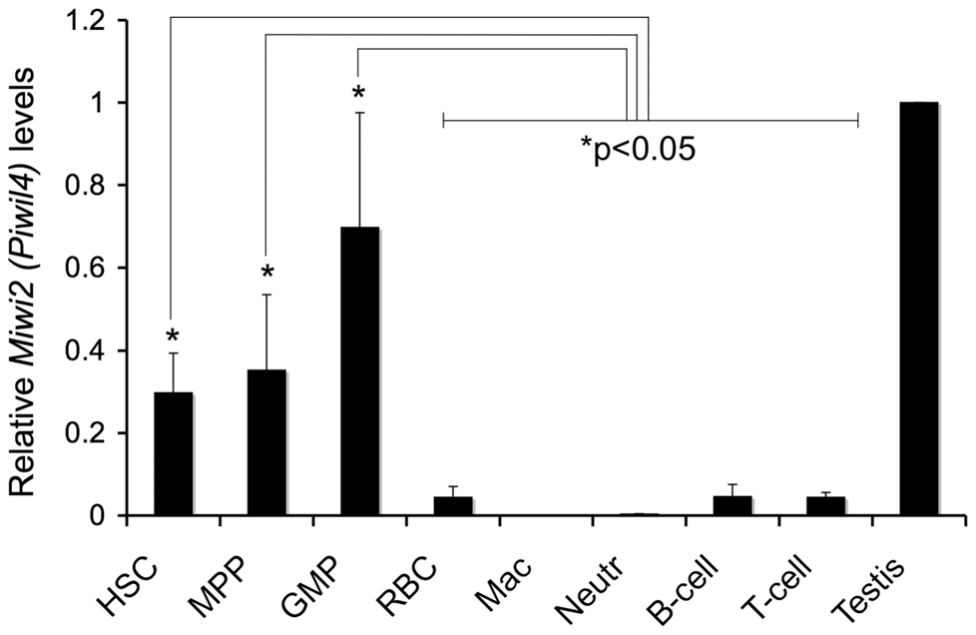
The mammalian *Miwi2* gene is expressed in hematopoietic stem and progenitor cells. Real-time PCR of FACS sorted young adult wild-type mouse bone marrow cells show expression of *Miwi2 (Piwil4)* at elevated levels in Lin^−^ cell types, hematopoietic stem cells (HSC), multi-potent progenitors (MPP) and granulocyte-macrophage progenitors (GMP), but at low or undetectable levels in Lin^+^ cell types, red blood cells (RBC), macrophages (Mac), neutrophils (Neutr), B-cells, and T-cells. Quantitative PCR samples were normalized to *Gapdh*. Data is the average of triplicate samples from combined bone marrow from three mice. As indicated, the difference between the stem/progenitor cell types and the differentiated cell types is statistically significant. *P values: HSC vs: MPP  = 0.667, GMP  = 0.078, RBC  = 0.012, Mac  = 0.006, Neutr  = 0.006, B-cell  = 0.012, T-cell  = 0.011; MPP vs: GMP  = 0.146, RBC  = 0.043, Mac  = 0.028, Neutr  = 0.029, B-cell  = 0.044, T-cell  = 0.042; GMP vs: RBC  = 0.015, Mac  = 0.012, Neutr  = 0.012, B-cell  = 0.015, T-cell  = 0.015. *P* values calculated using two-tailed, equal variance *t* test.

To begin our investigation, we examined the adult hematopoietic lineage in the bone marrow for expression of the three mouse *piwi* genes. Previous studies showed that the expression of *Miwi (Piwil1)* and *Mili (Piwil2)* is not detectable significantly above the background in hematopoietic cells, [6], [29]. Therefore, we focused on the *Miwi2 (Piwil4)* gene, the ortholog of *HIWI2*, which has previously shown to be the most ubiquitously expressed human *PIWI* homolog [18]. Using RT-PCR, we detected high levels of murine *Miwi2* in undifferentiated cell types, including immunophenotypic hematopoietic stem cells (HSC; Lin^-^cKit^+^Sca^+^CD150^+^CD105^+^), multi-potent progenitors (MPP; Lin^−^cKit^+^Sca^+^CD150^−^CD105^−^), and granulocyte-macrophage progenitors (GMP; Lin^−^cKit^+^Sca^−^CD150^−^CD16/32^+^), as compared to more committed cell types, red blood cells (RBC), macrophages (Mac), neutrophils (Neutr), B-cells, and T-cells (Fig. 1). These data are consistent with previously published results for human hematopoietic cells, and suggests that mouse piwi genes may have a regulatory function in undifferentiated cell types during normal hematopoiesis.

### Piwi genes are dispensable for steady-state hematopoiesis

**Figure 2:**
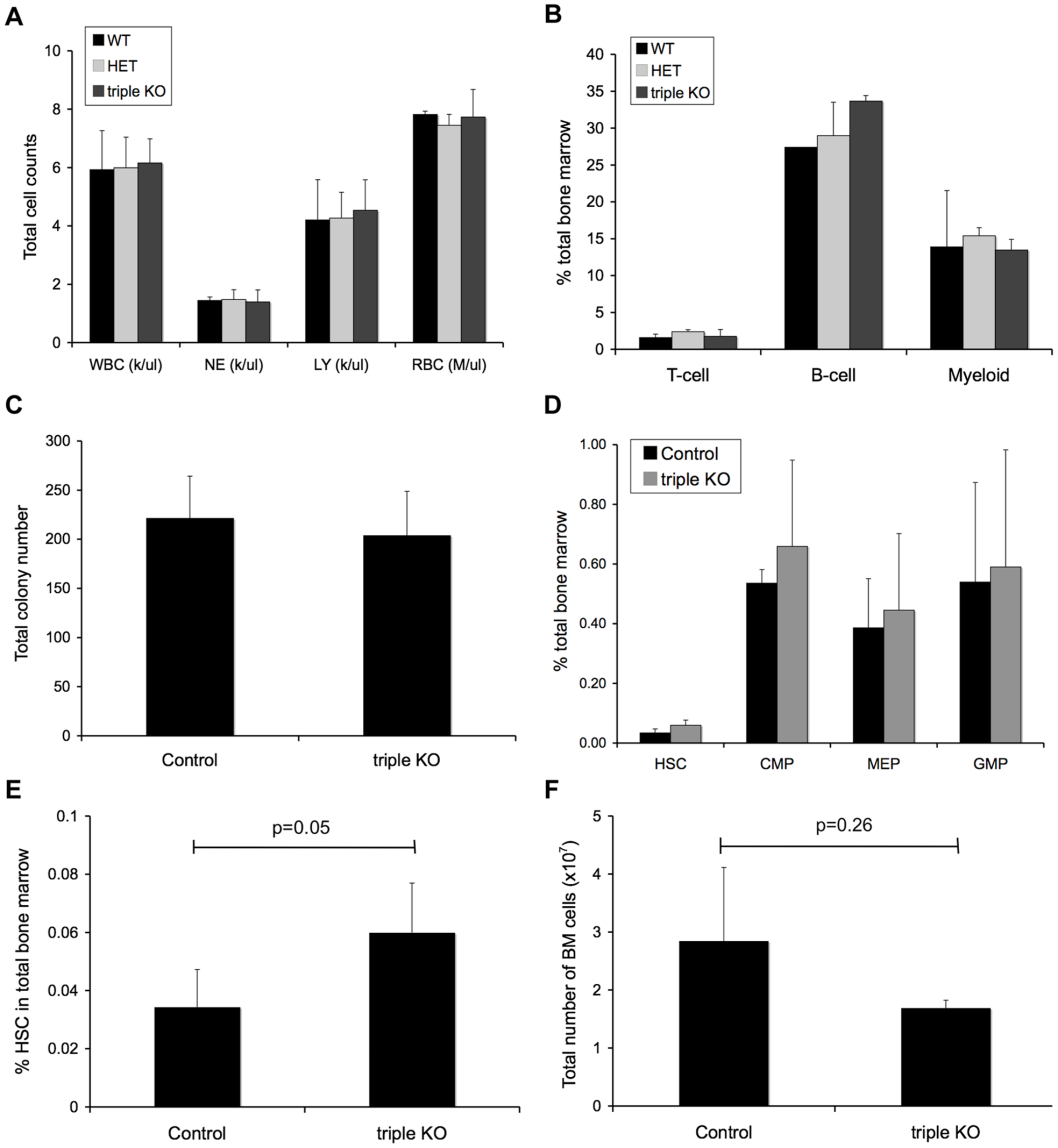
Deletion of all three piwi genes causes increased HSC frequency, but does not affect progenitor or committed cell type numbers. Piwi-triple-knockout mature blood cell numbers are unchanged compared to control mice (WT, HET), as determined by (A) Complete Blood Cell Count (CBC) for white blood cells (WBC), neutrophils (NE), lymphocytes (LY), red blood cells (RBC) and (B) FACS analysis of committed bone marrow cell types (A: WT, n = 3; HET, n = 4; triple KO, n = 4; B: WT, n = 2; HET, n = 3, triple KO, n = 3). (C) Colony Forming Unit (CFU) assays show similar total myeloid/erythroid progenitor-derived colony numbers in control and triple *Piwi* mutant bone marrow. Colony assays used total bone marrow from 3 individual mice for each genotype. Bone marrow samples were plated in triplicate and total colonies were counted at days 7–10. Data shown as the average total colony count for each genotype. (D) In the triple *Piwi* mutant, the percentage of bone marrow progenitors (CMP, MEP and GMP) are only moderately increased compared to control mice progenitors. (E) HSC percentages are significantly increased in *Piwi* triple knockout bone marrow. (F) Total bone marrow cellularity of the triple knockout mice is almost two-fold reduced compared to control mice. (C-F: Control, n = 4; triple KO, n = 4). *P* values calculated using two-tailed, unequal variance Student's *t* test.

Given the expression of *Miwi2* gene products in the hematopoietic system and the previously published influence of *Mili* overexpression on hematopoietic cell expansion [6], we investigated the potential function of piwi genes in maintaining HSCs and hematopoietic progenitors during hematopoiesis in mice. To avoid the complication of possible functional redundancy, we utilized a triple knockout mouse model whereby the three piwi genes are simultaneously deleted, leading to complete loss of piwi expression during development. In these triple-knockout mice, total peripheral blood leukocytes, erythrocytes, and platelets (not shown) are similar to those in wild-type and triple heterozygous littermates (Fig. 2A), suggesting that PIWI proteins are not needed for mature blood cell formation. Similarly, committed cell types isolated from the bone marrow of young adult mice showed no difference between piwi mutant and wildtype and heterozygous control animals (Fig. 2B). Additionally, myeloid colony forming unit (CFU) assays were performed to test for the presence of myeloid/erythroid progenitors in the bone marrow. Consistent with our FACS data above, we observed no difference in total colony numbers between the triple piwi mutant and wild-type control bone marrow (Fig. 2C).

To examine more specifically the effect of *Piwi* deletion in the hematopoietic stem and progenitor cell compartment, we analyzed HSC and progenitor percentages in mutant versus control bone marrow by FACS analysis. While progenitor populations for common myeloid progenitors (CMP), myeloid-erythroid progenitors (MEP) and GMP remained unaffected by the deletion of the three piwi genes (Fig. 2D), we did note an almost two-fold increase in the percentage of phenotypic HSC in the *Piwi* knockout mice (0.06%) as compared to the wildtype and heterozygous control mice bone marrow (0.03%, Fig. 2E). However, this difference was offset by a decrease in the average total bone marrow cellularity in the triple mutant as compared to the wild-type control mice (1.7×10^7^ HSC and 2.8×10^7^ HSC, respectively, Fig. 2F). Therefore, the total number of triple mutant HSCs in bone marrow remains similar to that of the wild-type with an average of 1×10^4^ HSCs in the triple mutant as compared to 8×10^3^ HSCs in control marrow. Overall, our data indicate that there is no significant influence of triple mutant on steady-state hematopoiesis.

### Triple piwi mutant HSCs can sustain hematopoiesis as assayed by competitive transplantation

**Figure 3:**
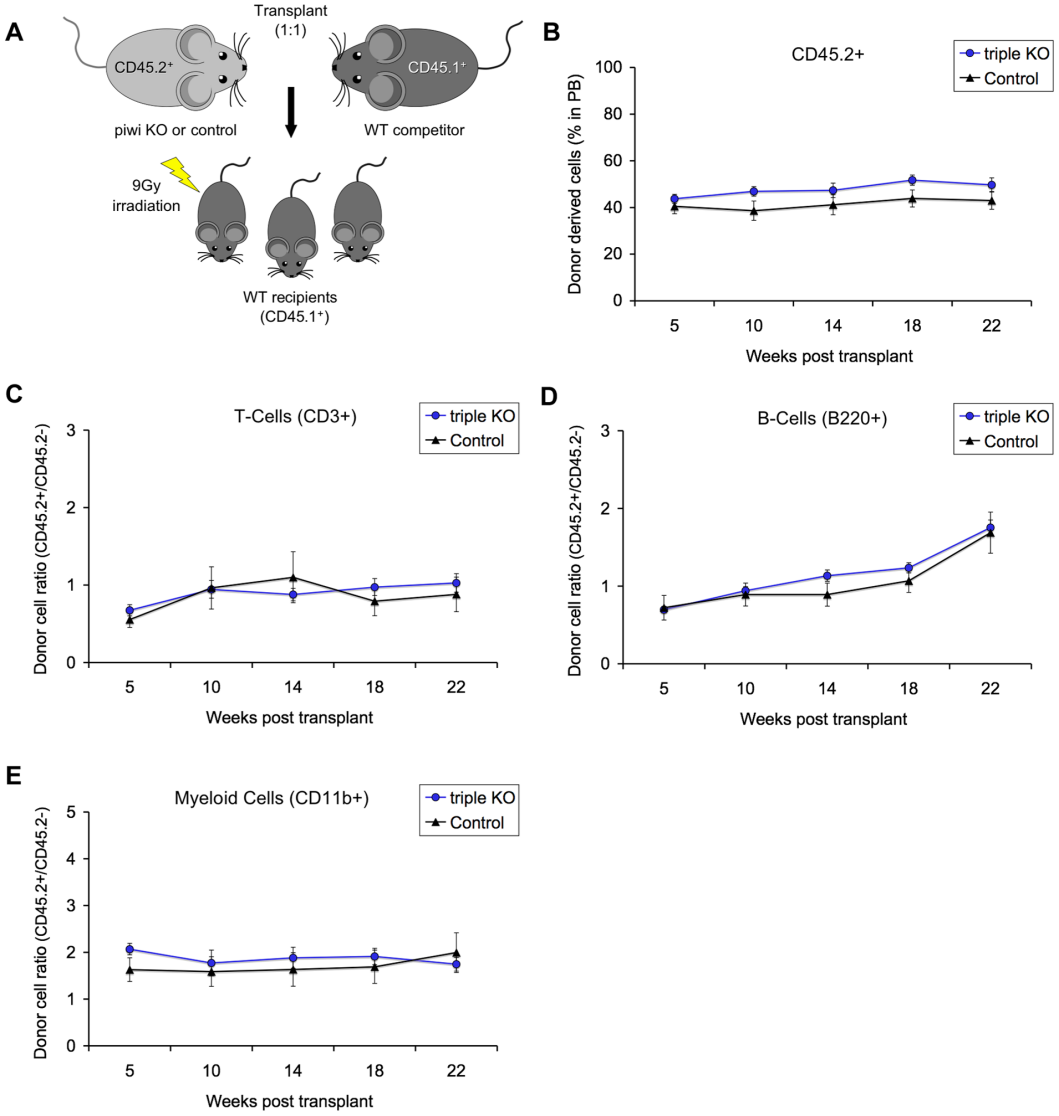
Piwi genes are not required for marrow HSC engraftment following irradiation. (A) Illustration of competitive transplantation scheme whereby the triple piwi mutant or control donor CD45.2^+^ bone marrow is mixed equally with competitor CD45.1^+^ bone marrow and injected into a cohort of lethally irradiated CD45.1^+^ wild-type recipient mice. (B) Percentages of donor-derived (CD45.2^+^) cells from the triple knockout (blue circle) and control (black triangle) donors in the peripheral blood of lethally irradiated CD45.1^+^ recipient mice, measured up to five months (22 weeks) after transplant. (C-E) Ratio of donor/recipient (CD45.2^+^/CD45.2^−^ ) derived cells within committed blood cell lineages, (C) T-cell (CD3^+^), (D) B-cell (B220^+^), and (E) myeloid cells (CD11b^+^). Data is the average of two independent experiments with 10 triple *Piwi* mutant injected recipients and 8 control injected recipients from each experiment. Each data point represents the mean ±SEM of 8–10 recipient mice, per donor genotype.

Because hematopoietic components do not always display overt phenotypes when disrupted under steady-state conditions and subtle differences could be masked by the strong homeostatic control mechanisms, we further investigated the function of triple mutant HSCs using a competitive transplantation assay (diagramed in Fig. 3A). We transplanted equal numbers of the triple mutant or control whole bone marrow, expressing the CD45.2^+^ antigen, together with wild-type CD45.1^+^ competitor bone marrow, into lethally irradiated CD45.1^+^ mice. This antigen marker system allows for tracking of hematopoiesis of donor cells within the transplanted hosts. We determined the proportion of donor-derived CD45.2^+^ cells in the recipient mice by analyzing peripheral blood up to 22 weeks post-transplantation. The triple mutant HSCs constituted a slightly elevated proportion of the blood (47%) compared to control donor-derived cells (39%) as seen by ten weeks following transplant (Fig. 3B). However, a continued expansion of the mutant CD45.2-derived cells did not occur, suggesting that PIWI deficiency does not confer any long-term repopulating advantage or disadvantage to HSC. We then assessed the competitiveness of the donor-derived cells in giving rise to various lineages, by looking at the ratio of CD45.2^+^/CD45.2^−^ cells within committed cell types of the blood system including T-cells (Fig. 3C), B-cells (Fig. 3D), and myeloid cells (Fig. 3E). For all time points assessed, the competitive repopulation ability and kinetics of the triple mutant was similar to the wild-type control bone marrow-derived cell types. To confirm establishment of mutant and control cells within the recipient bone marrow, CD45.2^+^ and CD45.2^−^ bone marrow cells were isolated by FACS from representative recipient mice and genotyped for individual *Piwi* genes. All mice showed expected genotypes (Fig. S1). Thus, triple deletion does not affect the ability of HSC for engraftment or in supporting normal hematopoiesis.

### The triple piwi mutant recovers normally following myeloablative injury

One of the key functions of HSCs is to replenish the lost cells following tissue injury [36]. To determine a role for piwi genes in HSC injury response, we injected mice with the widely used myeloablative agent 5-fluorouracil (5FU), 20 weeks after competitive transplantation (as described above), when homeostasis had long been re-established. We then used FACS analysis of peripheral blood cell types to assess the recovery process by the established *Piwi* mutant or control HSCs.

**Figure 4:**
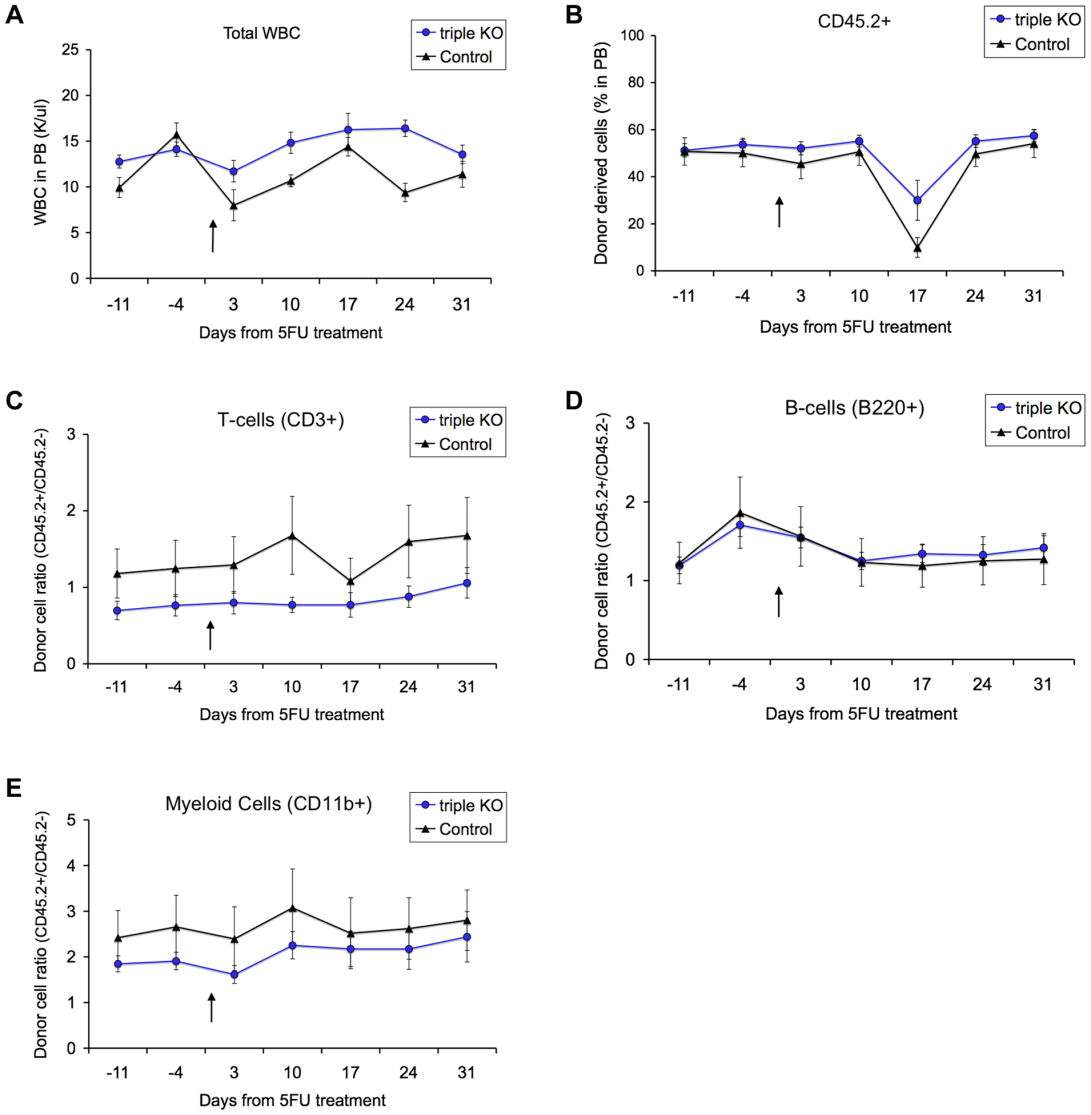
HSC myeloablative stress response is not affected by deletion of all piwi genes. (A) White blood cells (WBC) are responsive to 5FU in both piwi triple knockout (blue circle) and control (black triangle) donor-derived cells. (B) Piwi triple knockout (blue circle) and control (black triangle) donor-derived cells (CD45.2^+^) show similar response kinetics following 5FU, measured up to 31 days after 5FU treatment. (C-E) Ratio of donor/recipient (CD45.2^+^/CD45.2^−^ ) derived cells from *Piwi* triple knockout (blue circle) and control (black triangle) donors in mature cell populations of recipient peripheral blood. (C) T-cell (CD3^+^), (D) B-cell (B220^+^), and (E) myeloid cell (CD11b^+^) lineages. 5FU injection point is indicated as a black arrow on each graph. Data is the average of 10 triple *Piwi* mutant injected recipients and 9 control injected recipients. Each data point represents the mean ±SEM of 8–10 recipient mice, per donor genotype.

To confirm the efficiency of the 5FU treatment, Complete Blood Counts (CBC) analysis showed an expected drop in total white blood cell (WBC) numbers in both *Piwi* knockout and control-derived cells at day 3, with recovery observed by around day 14 (Fig. 4A), as was previously reported following 5FU exposure [37].

Analysis of peripheral blood donor-derived CD45.2^+^ *Piwi* mutant and control cells showed no difference in injury response to 5-FU treatment. A transient drop in donor cell percentages was observed for both the triple mutant and control-derived CD45.2^+^ cells at day 17 post-5FU with quick recovery noted by the next time point (Fig. 4B). This drop is likely due to a technical variation at this time-point. Within specific lineages, the recovery of B-cells and myeloid cells was unaffected by PIWI deletion (Fig. 4D and Fig. 4E, respectively). A slightly less dynamic response was noted for *Piwi* mutant-derived T-cells (Fig. 4C) compared to control-derived T-cells. However, there is a high amount of variability between individual mice within the control group and this may account for the observed fluctuations in T-cell numbers at day 10 and day 17 post-5FU. Overall, no significant difference was detected between the triple *Piwi* mutant and the wild-type control with respect to HSC injury response.

## Discussion

The expression of piwi genes in undifferentiated CD34^+^ cell types within human bone marrow[5] and the overexpression study of PIWI proteins in human and mouse leukemic cancer cells raised the possibility for a function of PIWI proteins in mammalian hematopoiesis. Our triple piwi knockout approach has allowed us to definitively address this question. We identified that one of the mouse piwi genes, *Miwi2 (Piwil4)*, is specifically expressed in immunophenotypically isolated HSCs and progenitors of adult bone marrow, but not in committed cell types. However, despite this expression, our findings indicate that murine PIWI proteins do not significantly contribute to the ability of HSCs to maintain hematopoiesis or the proliferation and differentiation of their progeny under homeostatic conditions in adult mice. Furthermore, piwi genes are dispensable for HSC recovery from competitive and replicative stress conditions.

In the absence of all three piwi genes, the number of phenotypic HSCs is only slightly increased compared to control mice. Additionally, the triple piwi mutant hematopoietic cells were able to maintain normal bone marrow progenitor cell populations and peripheral blood counts. This indicates that piwi-deficient HSCs are able to maintain a balanced hierarchy of downstream lineage committed progeny under homeostatic conditions.

We also found that following irradiation and in the presence of competitor cells, the triple mutant HSCs were functionally competent in homing to the bone marrow and in giving rise to all differentiated lineage cells of the peripheral blood system. Additionally, the triple mutant HSCs are able to appropriately recover from 5FU-induced myeloablative stress with similar kinetics to the control mice.

While the observed progression of hematopoiesis in the triple mutant mice indicates that piwi genes are not required for hematopoiesis, it does not mean that these proteins are involved in hematopoiesis. For example, Piwi proteins might work collectively with other hematopoietic regulators in a functionally redundant fashion, so that additional factors would need to be mutated simultaneously with the piwi genes in order to see a tractable effect on hematopoiesis. It is also possible that in order to detect a role for PIWI during hematopoiesis the mice may require a more significant physiological or environmental challenge that is not available within our assay constraints, when performed under stringent laboratory conditions.

While piwi genes are not needed to maintain normal hematopoiesis by our findings, their aberrant expression has been correlated with cancerous cell states in many different tissue types. Chen and colleagues demonstrate, directed overexpression of *Mili* (*Piwil2*) can drive proliferation of normal bone marrow cells in culture [6]. Another recent study reported that the progressive differentiation of HSCs to restricted lineages is characterized by tractable chromatin remodeling at lineage specific gene loci [38]. Because cancer cells are characterized by hyper-proliferation and given the known role of Piwi proteins and their partner piRNAs in epigenetic regulation [31], [39]–[41], it would be interesting to further examine a functional role for piwi genes during malignant hematpoietic transformation and leukemic progression, with the possibility that aberrant PIWI expression in the blood system can drive progenitors to uncontrolled expansion, at least in part, through an epigenetic mechanism.

## Supporting Information

Figure S1 **Genotyping of transplanted bone marrow cells in recipient mice.** FACS sorted CD45.2^+^ and CD45.2^−^ bone marrow cells isolated from the hind limbs of representative transplant recipient mice, representing *Piwi* triple knockout and control donor cohorts, show expected genotypes for *Miwi*, *Mili*, and *Miwi2* alleles, with wild-type and heterozygous expression in CD45.2^+^ cells from control donor mice (601 and 603) and knockout alleles for CD45.2^+^ cells from triple mutant donor mice (610 and 613). CD45.2^−^ competitor cells and wild-type mouse tail DNA show expected wild-type alleles for all three genes. For isolation, nucleated bone marrow cells were incubated with CD45.2-FITC antibody and sorted on a LSRII (BD). Genomic DNA was then purified from CD45.2^+^ and CD45.2^−^ cells and used for PCR.
(TIF)
