# Supplementary figures and images for: Piwi Genes Are Dispensable for Normal Hematopoiesis in Mice
Source: PLoS One. 2013 Aug 23;8(8):e71950. doi: 10.1371/journal.pone.0071950 (PMC3751959; doi:10.1371/journal.pone.0071950)

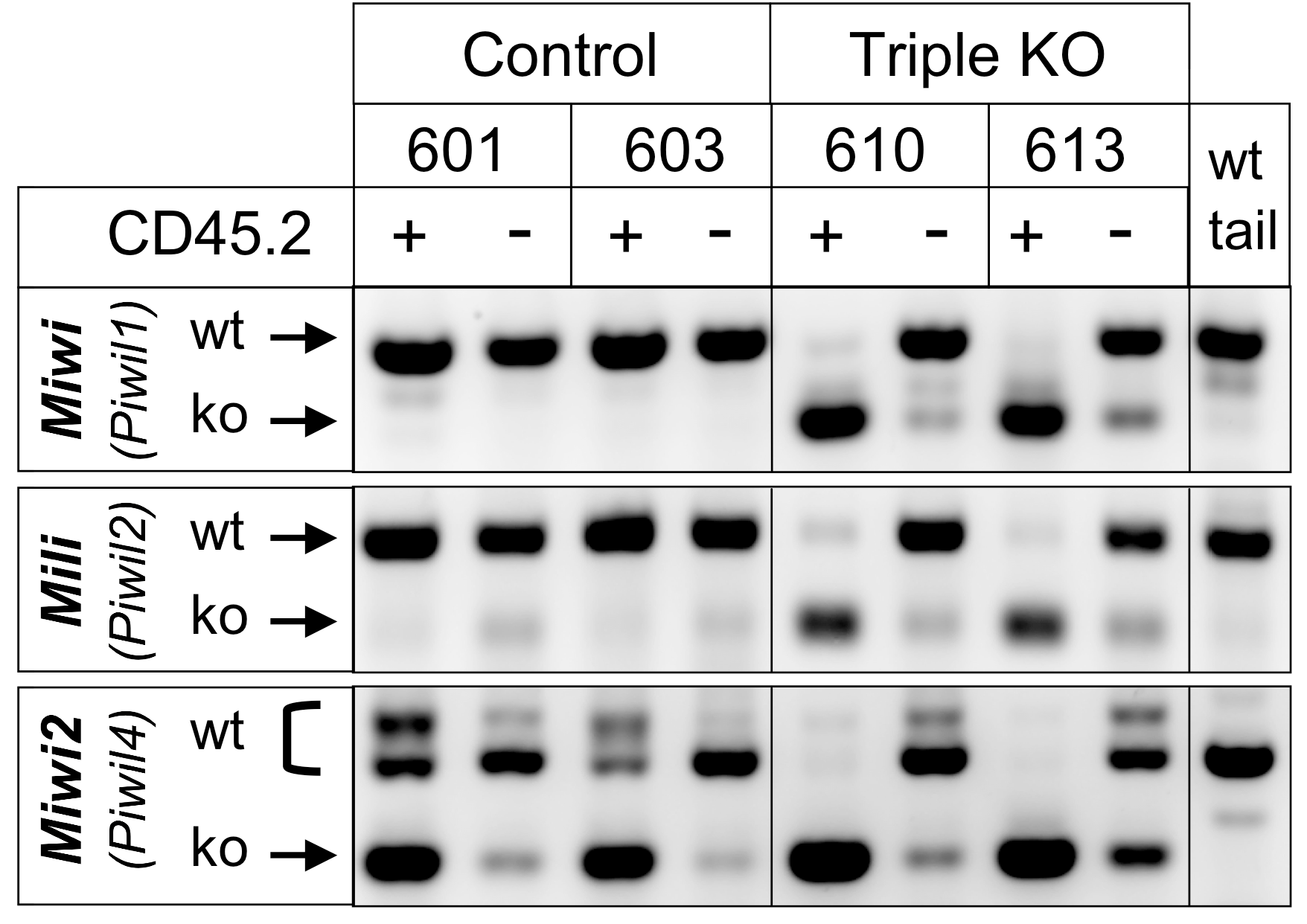

Supplement: Figure S1 — Genotyping of transplanted bone marrow cells in recipient mice. FACS sorted CD45.2+ and CD45.2− bone marrow cells isolated from the hind limbs of representative transplant recipient mice, representing Piwi triple knockout and control donor cohorts, show expected genotypes for Miwi, Mili, and Miwi2 alleles, with wild-type and heterozygous expression in CD45.2+ cells from control donor mice (601 and 603) and knockout alleles for CD45.2+ cells from triple mutant donor mice (610 and 613). CD45.2− competitor cells and wild-type mouse tail DNA show expected wild-type alleles for all three genes. For isolation, nucleated bone marrow cells were incubated with CD45.2-FITC antibody and sorted on a LSRII (BD). Genomic DNA was then purified from CD45.2+ and CD45.2− cells and used for PCR. (TIF) [file pone.0071950.s001.tif]
